# First detection of D181 genotype of infectious bronchitis in poultry flocks of Morocco

**DOI:** 10.1186/s12985-024-02539-z

**Published:** 2024-10-28

**Authors:** Mouahid Mohamed, Bidoudan Yassmina, Regragui Rim, El Kaouani Mouna, Fellahi Siham

**Affiliations:** 1 Mouahid’s Veterinary Clinic, Temara, 12000 Morocco; 2 Department of Veterinary Pathology and Public Health, Agronomy and Veterinary Institute Hassan II, Rabat-Instituts, Rabat, BP 6202, Morocco

**Keywords:** Avian coronavirus, Infectious bronchitis virus, IBV, D181, Poultry, Morocco

## Abstract

**Background:**

This paper reports the first pathological and molecular characterization of the novel variant of infectious bronchitis virus (IBV) D181 in poultry flocks in Morocco and Africa.

**Methods:**

The study includes six poultry farms, involving three flocks of layers aged between 28 and 67 weeks and three broiler flocks aged 27, 39 and 42 days from different regions of Morocco. In all affected layer flocks, a severe drop in egg production with poor eggshell quality was reported. Necropsy of dead birds was carried out, and samples of trachea, lungs, oviduct, ovaries, and kidneys were fixed in 10% neutral buffered formalin for histopathologic examinations, while other portions were stored at -20 °C for molecular analysis. Real time RT-qPCR for IBV gene group was performed, and IBV variants were identified. Partial S1 gene sequences were amplified by conventional RT-PCR, sequenced, and aligned for phylogenetic and amino acid similarity analysis.

**Results:**

Necropsy of dead birds revealed misshapen and hemorrhagic ovarian follicles with an edematous oviduct and severe reaction in the cecal tonsils. A caseous material accumulation in the sinus was noted in few birds. In contrast, the broiler flocks exhibited respiratory clinical signs such as difficulty in breathing, sneezing, tracheal rales, watery eyes and lethargy, associated with a decrease in feed consumption. Mortality in broiler ranged from 2 to 15%. Histopathological analysis of samples showed a lympho-plasmocytic inflammation in the oviduct, trachea, and lungs. Individual necrosis of epithelial cells, with sloughing of the bronchial epithelium and accumulation of desquamated cells with mucus in the airways, was observed in some birds. Partial S1 gene sequencing and phylogenetic analyses showed that the Moroccan strains were very closely related to D181 strains isolated in Dutch layers and breeders in 2018. Nucleotide sequence identities reached 90.9–95% with the Dutch isolates (strain CK/NL/D181/2018).

**Conclusion:**

Our sequencing results demonstrate for the first time that the D181 IBV genotype is circulating in Moroccan poultry. These findings justify permanent monitoring of circulating strains in order to appropriately adjust vaccination strategies to align with the evolving field situation.

## Introduction

Infectious bronchitis (IB) is a highly contagious disease in chickens with worldwide distribution [1, 2]. It affects the respiratory epithelial cells, as well as the urinary, reproductive and intestinal tracts of birds of various ages [3]. IB outbreaks result in heavy economic losses, primarily due to a drop in egg production, deterioration in eggshell quality, decreased hatchability, increased feed conversion ratio, and increased condemnation at slaughterhouses [4]. The etiological agent is an RNA virus belonging to the family of *Coronaviridae* genus *Gammacoronavirus*, and is specific to the chicken (*Gallus gallus*) [5].

The virus genome consists of a single stranded, positive sense, enveloped RNA of approximately 27,6 kilobases (Kb) in length [3, 6]. The spike protein (S) is translated as a precursor protein (S0) and later cleaved into the amino-terminal S1 and carboxyl-terminal S2 subunits, which remain attached. The S1 protein is responsible for virus attachment and entry into host cells and plays an essential role in tissue tropism, induction of protective immunity, virus neutralization, and serotype specificity [7], while the S2 subunit is involved in cells entry. Infectious bronchitis virus (IBV) is extremely difficult to control due to its high genetic diversity, driven by a significant mutation rate [8, 9]. Furthermore, its evolution through spontaneous mutations and recombination generates genetic and antigenic variants that can be unaffected by existing vaccines [1]. IBV can also evade the host’s immune response, causing disease in chickens even after vaccination [10]. In Moroccan poultry farms, the first detection of IBV was reported by El Houadfi and Jones [11]. The isolates were serologically related to the Massachusetts (Mass) serotype and a novel genotype designated as Moroccan ‘‘G” which is closely related to 4/91 (793B) serotype, with a potentially common origin [12, 13]. An epidemiological survey conducted between 2010 and 2014 by Fellahi et al. [14] showed the emergence of a novel strain of the Italy02 genotype, with a prevalence of 32% co-circulating with Mass and 4/91 serotypes at a prevalence of 66% and 2% respectively. Recently, between 2019 and 2022, the prevalence of genotypes has shifted, with a re-emergence of the 793B variant (54%), followed by Italy 02 (27%), the new genotype “Lotfi” (14%) and only 5% for the Mass genotype [15–17]. In this paper, we report and describe the first detection of the novel IBV variant D181 (serotype 2) in Moroccan layers and broilers. The authors recommend considering this variant as an important differential diagnosis for egg drop and eggshell deterioration in layer farms.

## Materials and methods

### Case history

**Fig. 1:**
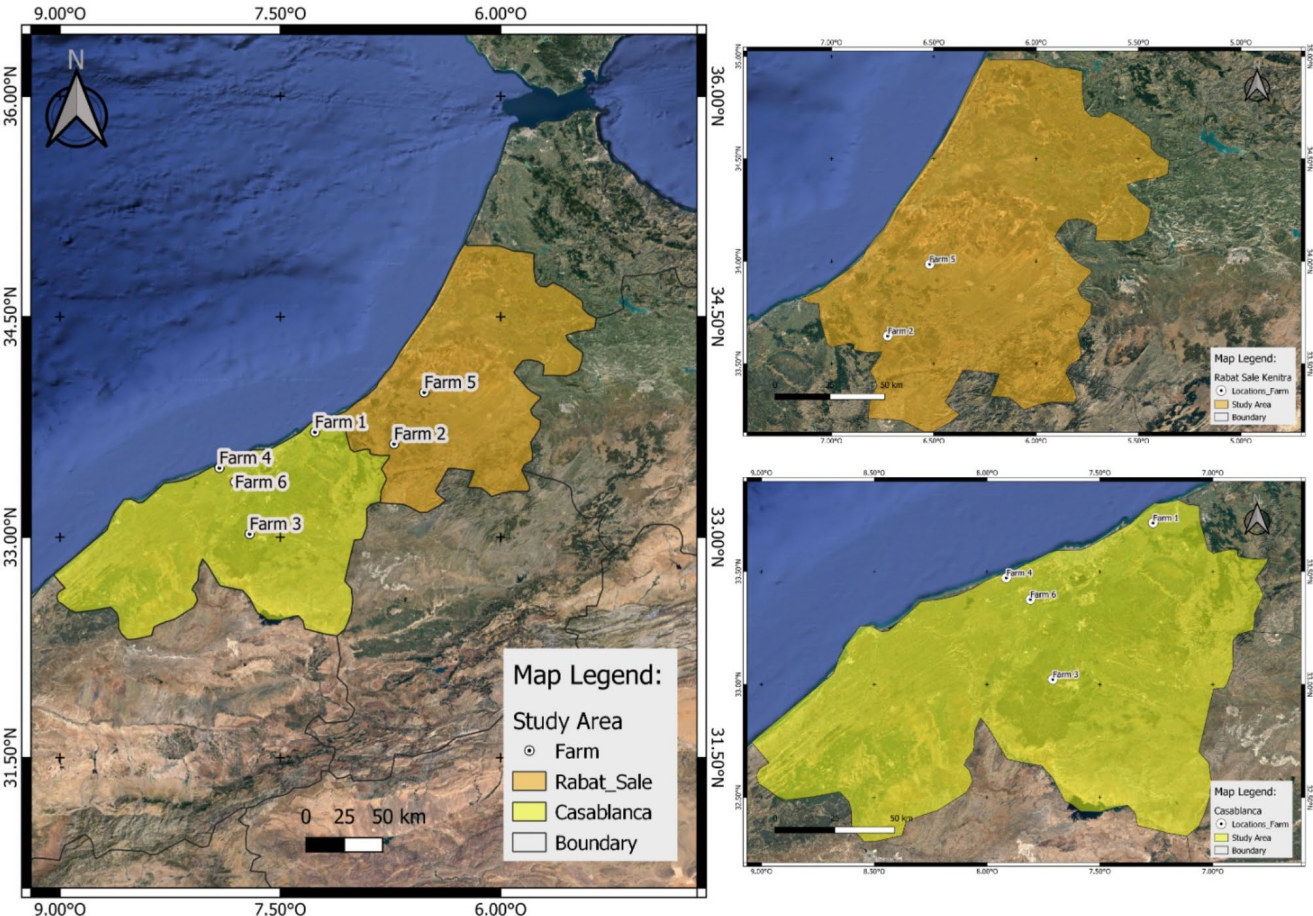
Geographic location of the IBV D181 affected flock

Between March and June 2022, six poultry farms (three broiler and three layer farms) from the Casablanca-Settat and Rabat regions of Morocco (Fig. 1) exhibited a decrease in feed consumption, severe respiratory signs including sneezing, coughing, rales and gasping for broilers, and a decrease in feed consumption in addition to a drop in egg production and altered eggshell quality in layers. Details of the flocks are presented in Table 1. All layer flocks were vaccinated against IB virus using the Mass strain at 1 day, 7 and 12 weeks of age, in addition to the 793B vaccine strain at 1 day, 12, and 17 weeks of age. Additionally, all flocks were vaccinated with inactivated IB vaccine at point of lay. The three broiler flocks were vaccinated only at the hatchery with Mass strain.

**Table 1:** Data of affected flocks with the novel IBV variant D181

Farm	Flock	Production type	Age (w/d^*^)	Population size/Flock	Clinical signs	Region	Mortality Rate (%)	Concurrent infections
1	F1	Layers	32w	38,000	Drop in egg production Poor eggshell quality	Casablanca	< 0.1	--
	F2	Layers	32w	38,975	Drop in egg production Poor eggshell quality Respiratory signs Slight increase in mortality	Casablanca	< 0.1	*M. Gallisepticum* *M. Synoviae*
	F3	Layers	32w	38,000	Drop in egg production Poor eggshell quality Respiratory signs Slight increase in mortality	Casablanca	< 0.1	*M. Gallisepticum* *M. Synoviae*
	F4	Layers	32w	38,000	Drop in egg production Poor eggshell quality	Casablanca	< 0.1	--
2	F5	Layers	65w	26,600	Drop in egg production Poor eggshell quality	Rabat	< 0.1	--
	F6	Layers	67w	36,700	Drop in egg production Poor eggshell quality	Rabat	< 0.1	--
3	F7	Layers	28w	196 117	Drop in egg production Poor eggshell quality	Casablanca	< 0.1	--
4	F8	Broilers	42d	39,000	Respiratory signs	Casablanca	15	--
5	F9	Broilers	27d	18,000	Respiratory signs	Rabat	4	--
6	F10	Broilers	39d	15,000	Respiratory signs	Casablanca	2	--

^*^w = weeks ; d = days

### Necropsy and sample processing

Necropsies were performed on dead birds from each flock to access gross pathological lesions. Tracheal swabs, as well as samples of trachea, lung, oviduct, spleen, and cecal tonsils were collected from affected birds for further laboratory investigations. Samples were immediately placed on ice in sterile transport minimum essential medium (MEM) containing 5% antibiotics (20,000IU/ml penicillin, 10,000 µg/ml streptomycin, and 5000 µg/ml kanamycin) and sent to the Avian Pathology Unit laboratory at the Agronomy and Veterinary Hassan II Institute for analysis. Each sample was homogenized in Dulbecco’s Modified Eagle’s Medium (DMEM). Tissue suspensions were centrifuged at 8000 g for 20 min at 4 °C. Then, 500 µl of supernatant was clarified at 12,000 g for 1 min and processed immediately. Samples of the trachea, lungs, ovaries, oviduct, and kidneys were also placed in a 10% neutral buffered formalin. Sections of 5 μm were cut and stained with hematoxylin and eosin (H&E) for histopathology examination.

### Differential bacterial and molecular diagnosis

For layer farms, an initial screening was conducted for avian pathogens presenting with similar clinical and pathological presentations. The molecular screening included Newcastle disease virus (PMX-1), low pathogenic avian influenza subtype H9N2 (LPAIV H9N2), infectious laryngotracheitis virus (ILT), avian pneumovirus (AMPV), avian encephalomyelitis virus (AEV), avian coronavirus (IBV), and *Mycoplasma gallispeticum* and *Mycoplasma Synoviae.* For this purpose, combined RNA/DNA was extracted from sampled organs (trachea, lungs, spleen, cecal tonsil, and oviduct) using the Kylt^®^ RNA/DNA Purification kit (AniCon Labor GmbH, Emstek, Germany) following the manufacturer’s instructions, followed by one-step real time PCR using the Brilliant III Ultra-Fast qRT-PCR master mix (Agilent Technologies, Waldbronn, Germany) for virus detection. Table 2 presents the targeted genes, specific primers, and probes used for each virus. Quantitative PCR was carried out in a 20 µl reaction volume, with thermal cycling on the ABI 7500Fast (Applied Biosystems, Foster City, CA, USA). The process consisted of 10 min at 50 °C, followed by an initial denaturation at 95 °C for 3 min, and 40 cycles of 95 °C for 12 s, 60 °C for 15secondes. A Ct value ≤ 35 was considered positive.

**Table 2:** Primers and probes used for the molecular diagnosis of pathogens

Avian pathogens	Target gene	Primers and probes	Reference
PMX-1	Matrix (M)	M + 4100 5’AGTGATGTGCTCGGACCTTC-3’ M-4220 5’-CCTGAGGAGAGGCATTTGCTA-3’ M + 4169 5’-(FAM) TTCTCTAGCAGTGGGACAGCGC (TAMRA)-3’	[18]
LPAIV H9N2	Matrix (M)	H9F : 5’ ATGGGGTTTGCTGCC 3’ H9R : 5’ TTATATACAAATGTTGC AC (T) CTG-3’ Probe : H9probe : 5’ FAM-TTCTGGGCCATGTCCAATGG-TAMRA 3’	[19]
ILT	Glycoprotein C (gC)	ILTVgC U771 : 5’-CCT TGCGTTTGAATTTTTCTGT-3’ ILTVgCL873 : 5’-TTCGTGGGTTAGAGGTCTGT-3’ ILTVprobe817 :5’-FAM-CAGCTCGGTGACCCCATTCTA-BHQ1-3’	[20]
aMPV	N gene of aMPV-A and -B	aMPV-A-F: GGGAGCAATGGTTAGGGATAAA aMPV- A-R TGAGGGCACCAATGCATAATA aMPV-A-probe Cy5 : AATAACGGGAGCATCCAAGGCAGA- BHQ1 aMPV-B-F : CAAGCATGCAATCCTTGATGA aMPV-B-R : GTGGATACCTTTGGCTGTAGTT aMPV-B-probeFAM-GGGTGTGATAGCAGTTGTAGCACCA-MGBNFQ	[21]
IBV	5’ -UTR	IBV5 GU391 (5-GCT TTT GAG CCT AGC GTT-3) IBV5 GL533 (5-GCC ATG TTG TCA CTG TCT ATT G-3) G probe (5-FAMCAC CAC CAG AAC CTG TCA CCT C-BHQ1-3	[22]

For each positive IBV sample, an additional real time PCR for variant screening was performed using Kylt^®^ kits, including the 4/91, Italy 02, Arkansas, Qx, D1466, D274, Variant 2, IB80, and Massachusetts variants. For MG/MS and encephalomyelitis virus detection, Kylt^®^MGS triplex Kit and Kylt^®^avian encephalomyelitis virus (AniCon Labor GmbH, Hoeltinghausen, Germany) were used according the manufacturer’s instructions. The broiler chicken flocks were also investigated for LPAI H9N2, PMX-1, and AMPV, in addition to IBV and its variants.

Additionally, bacteriological testing was performed to rule out bacterial infections. However, these tests were not carried out on broiler samples, therefore, the health status of those birds in relation to bacterial infections remains unknown.

### Avian coronavirus RNA extraction and one-step real time RT-PCR

Total viral RNA was extracted from affected organs and tracheal swabs using a Kylt^®^ RNA/DNA Purification kit (AniCon Labor GmbH, Emstek, Germany), following the manufacturer’s protocol. Each sample of extracted RNA was recovered and subjected to real time RT-PCR amplification using primers described by [22]. The amplification was carried out using Invitrogen kit SuperScript^®^ III Platinum one-step qRT-PCR, (Life Technologies, USA). The reaction volume contained 12.5 µl 2× RT-PCR buffer mix, 0.5 µl MgSO4 (50mM), 0.5 µl Rox (25mM), 4.75 µl nuclease free water, 0.5 µl primers (10 µM), 0.25 µl (10 µM) and 5 µl RNA template. The reaction was conducted in an Applied Biosystems thermocycler (Foster City, CA) with the cycling conditions of 50 °C for 5 min, followed by 95 °C for 2 min, and 40 cycles of 95 °C for 3 s and 58 °C for 30 s.

### Virus isolation

To maximize viral load detection by conventional RT-PCR and for S1 gene sequencing, isolation in SPF eggs was attempted for positive samples with Ct values greater than 35 among the 10 positive flocks. Conventional RT-PCR analysis revealed that S1 PCR products were obtained for six isolates derived from three layer farms and three broilers flocks. A 0.2 ml sample of each organ (Trachea, lung and oviduct) and/or the supernatant of tracheal swab of five live birds was homogenized and inoculated individually into 10-day-old, specific pathogen free (SPF) embryonated eggs. Briefly, the eggs were mirrored, and the air chamber was delimited. The viral inoculum was prepared by mixing 0.2 ml of the viral suspension with 0.6 ml of sterile PBS and 0.2 ml of antibiotic OXY-Kel 20 L.A (Oxytetracycline). The mixture was injected via allantoic cavity route using a sterile needle into the air chamber. After a 7 days incubation period, the eggs were chilled, and the allantoic fluids were harvested, with specific embryonic lesions and mortalities recorded.

### RT-PCR and partial S1 sequencing

RT-PCR was performed in two steps. First, retro-transcription was conducted using High Capacity cDNA Reverse Transcription Kit (Applied Biosystem, Thermo Fisher Scientific, CA). Briefly, 10 µl of DMSO-treated viral RNA was mixed with 2 µl of 10x RT Random Primers, 0.8 µl of 25x dNTP mix, 2 µl of 10X-RT Buffer, 1 µl of the Multiscribe RT and 4.2 µl of RNase-Free dH2O, to total 20 µl. The mixture was incubated at 25 °C for 10 min and 37 °C for cDNA synthesis, and then the reaction was stopped at 95 °C for 15 min. The PCR reaction was performed with 35 cycles at 94 °C for 45 s, 48 °C for 45 s, and 72 °C for 1 min, with a final incubation at 72 °C for 10 min. PCR products were visualized on a 1.5% agarose gel and Thermo Scientific GeneRuler 100 pb DNA ladder (ThermoFisher, USA) was used as a molecular weight marker to estimate the size of the PCR products. The primers used for the amplification and sequencing of part of the S1 gene (hypervariable region 3) have been described by Jones et al. [23]. Initially, a detection PCR was used to amplify a 650 bp sequence, followed by a nested PCR to amplify a fragment of 392 bp.

### DNA sequencing

Six selected RT-PCR products (392 bp) generated from the nested RT-PCR were purified using the Gene Clean Kit (ExoSAP-IT), according to the manufacturer’s recommendations, and sequenced using the primers SX3 + and SX4- [24]. Nucleotide sequence determination was performed using the BigDye^®^ Terminator v1.1 Cycle. Sequencing kit, followed by purification using the Big Dye X Terminator Purification Kit.

### Nucleotide sequence analysis

Assembly and analysis of sequence data were carried out using BioEdit Software version 5.0.9 [25]. The open source BLAST program [26] was used for sequence comparison. Nucleotide and deduced amino acid sequences were aligned using ClustalW and MEGA software Version 6.0 [27]. Phylogenetic analysis and tree construction for the S1 glycoprotein were generated using the maximum likelihood (ML) method with 1,000 bootstrap replicates with the MEGA software Version 5.05 program [28], and bootstrap values above 50 were labeled on major tree branches for reference.

## Results

### Clinical characteristics of D181 IBV isolates

From March to June 2022, five D181 isolates were collected from two broilers flocks and three layer farms from different regions in Morocco. Two farms of laying hens, comprising four and three flocks aged 32 weeks and 67 weeks, respectively, reported a drop in egg production and altered eggshell quality. One flock of laying hens aged 28 weeks exhibited a drop in egg production ranging from 6 to 34%, with a severe drop in feed intake (Table 1). Moreover, the three broilers flocks, aged 27, 39 and 42 days, showed respiratory clinical signs such as difficulty in breathing, sneezing, tracheal rales, watery eyes, and lethargy, associated with a decrease in feed consumption. Mortality rates ranged from < 0.1–15%. Bacteriological tests were negative for all tested flocks. Additionally, PCRs tests for infectious laryngotracheitis virus, avian encephalomyelitis virus, Newcastle disease virus, avian influenza virus subtype H9N2, and pneumovirus were all negative for both broiler and layer flocks. On the other hand, *Mycoplasma* species were detected in tracheal swabs from two flocks on the first farm with mean Ct values of 25.15 and 28.96 for *Mycoplasma synoviae* and 24.37 and 19.9 for *Mycoplasma gallisepticum* among three sampled pools (Table 1). Moreover, PCR tests for avian coronavirus were positive for all the flocks, including both layers and broilers, with Ct values ranging from 29.2 to 35.4. However, the molecular screening was negative for all tested variants (4/91, Italy 02, Arkansas, Qx, D1466, D274, Variant 2, IB80, Massachusetts).

### Post-mortem examinations

**Fig. 2:**
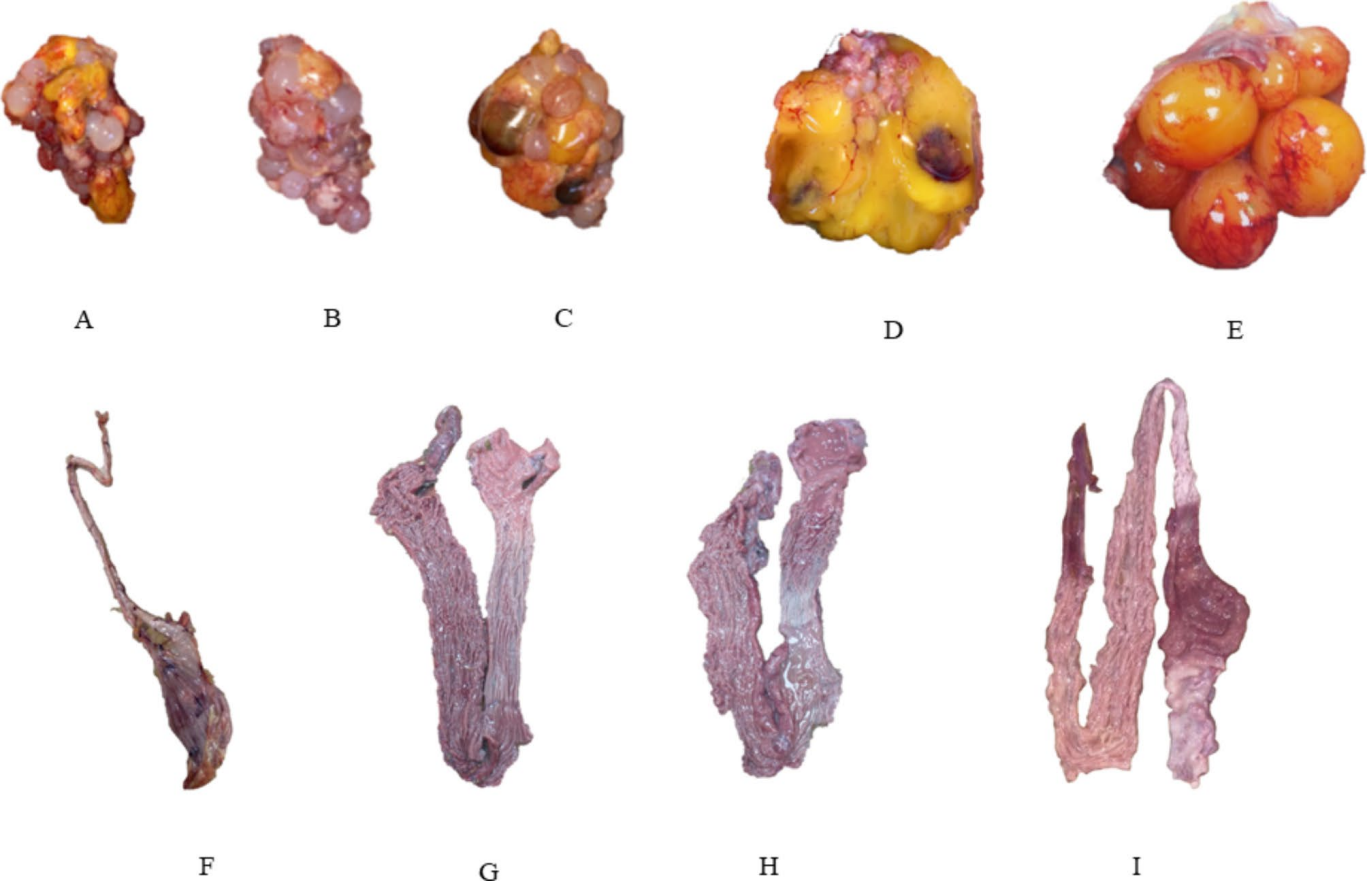
Gross lesions in necropsied laying hens. (**A-C**) Severe atrophy of the ovaries. (**D**) Misshapen ovarian follicles with hemorrhage. (**E**) normal ovary of a laying hen. (**F**) Atrophy of the oviduct. (**G-H**) Edema of the oviduct, (**I**) Normal oviduct

Necropsy examinations revealed that all broilers flocks (F8, F9 and F10) exhibited mucosal thickening, with serous or catarrhal exudates in the nasal passages, and congested or hemorrhagic tracheas and lungs. In contrast, birds of the first six layers flocks (F1 through F6) showed severe edema and congestion of the oviduct with misshapen and hemorrhagic ovarian follicles, and hemorrhage in the cecal tonsils (Fig. 2). A few birds of F2 and F3 had caseous material in the nasal cavities and the sinus. Birds of the last layer flock (F7) mainly presented atrophy of the reproductive tract (oviduct and ovaries), with only two out of eight necropsied birds showing misshapen ovarian follicles (Fig. 2).

### Microscopic lesions

**Fig. 3:**
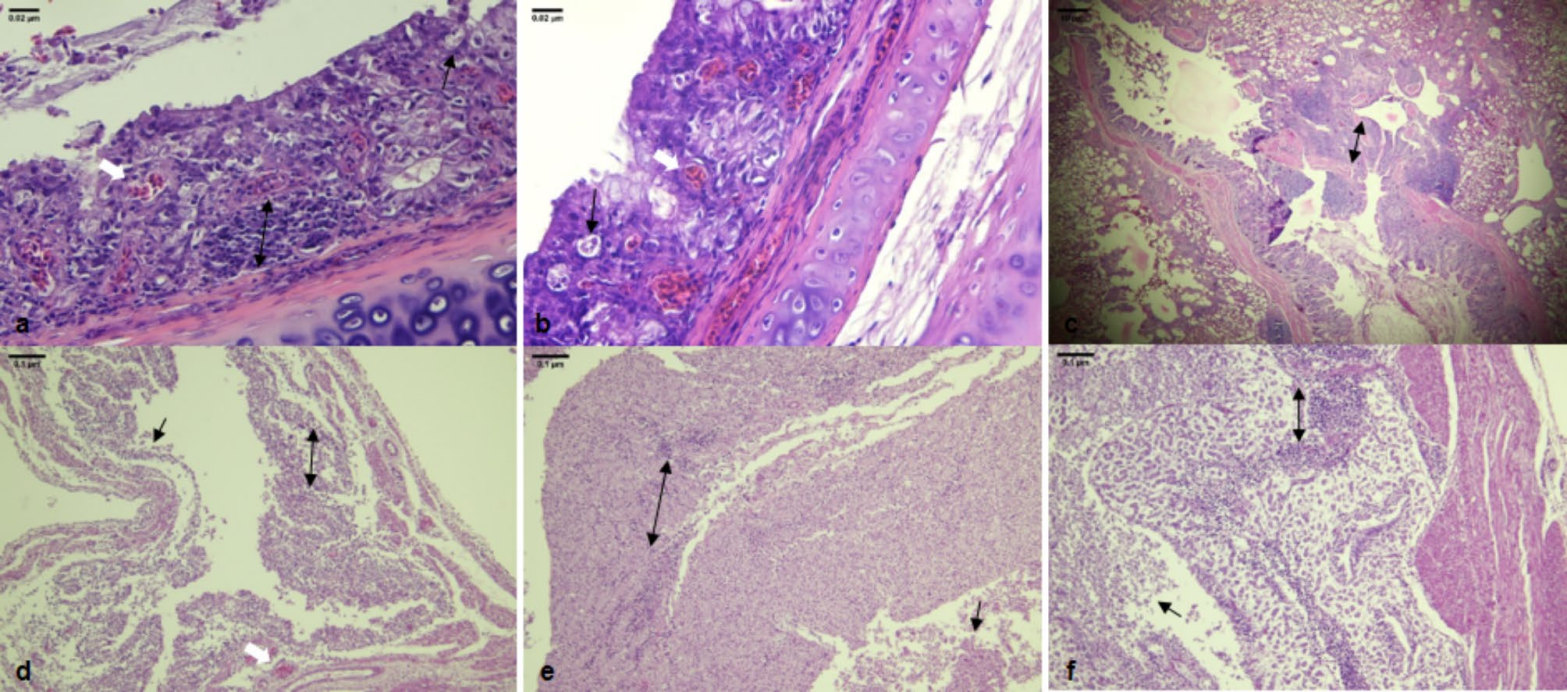
Histopathological lesions associated to the infection with the novel D181 genotype in laying hens. (**a-b**) **Trachea** with severe extensive loss of cilia and individual cell necrosis (**black arrow**), congestion **(white arrow**) and lympho-plasmocytic infiltration in the lamina propria (**double-headed arrow**). (**c**) **Lung** showing multifocal to extensive lymphocytic infiltration of the bronchial wall with accumulation of mucus and desquamated epithelial cell in the bronchial lumen. (**d-f**) **Oviduct** with a multifocal lympho-plasmocytic infiltration (**double-headed arrow**), focal mucosal necrosis and degeneration with desquamation of epithelial cells in the lumen (**black arrow**), and severe interstitial dilation and edema in the (**d**) infundibulum (**e**) isthmus and (**f**) the shell gland

Histopathological examination revealed lympho-plasmocytic bronchitis and tracheitis, with lymphocytic infiltration in the lamina propria and diffuse loss of cilia. Individual necrosis of epithelial cells was noted in birds from the flocks F7, along with the accumulation of desquamated epithelial cells and mucus in the bronchial lumen. Lympho-plasmocytic salpingitis was detected in birds of all layer flocks, with more severe lesions in the isthmus and shell gland (Fig. 3). No lesions were detected in the kidneys or ovaries of any examined birds.

### RT-PCR and sequencing of the S gene

**Fig. 4:**
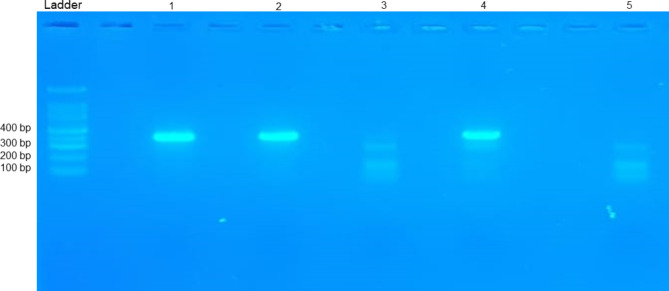
Agarose gel electrophoresis for the 392 bp amplified fragment of the S1 gene. Ladder line: 100 pb DNA Ladder; Lanes 1 and 2 positive samples; Lane 3: negative sample, Lane 4: positive control; Lane 5: negative control

IBV is classified into genotypes based on the characteristics of its spike glycoprotein (S) gene and protein [29]. To detect the virus, samples were first amplified by real-time RT-qPCR and then amplified by conventional RT-PCR for sequencing of the partial S1 gene, which carried the antigenicity of the virus (Fig. 4). Fragments of 392 bp of the gene coding for a portion of the S1 gene were sequenced using the corresponding primers, allowing the detection of five strains of the D181 genotype. The accession numbers of the Moroccan D181 strains for farms F1 to F5 are OR708717 to OR708721.

### Phylogenetic analysis

**Fig. 5:**
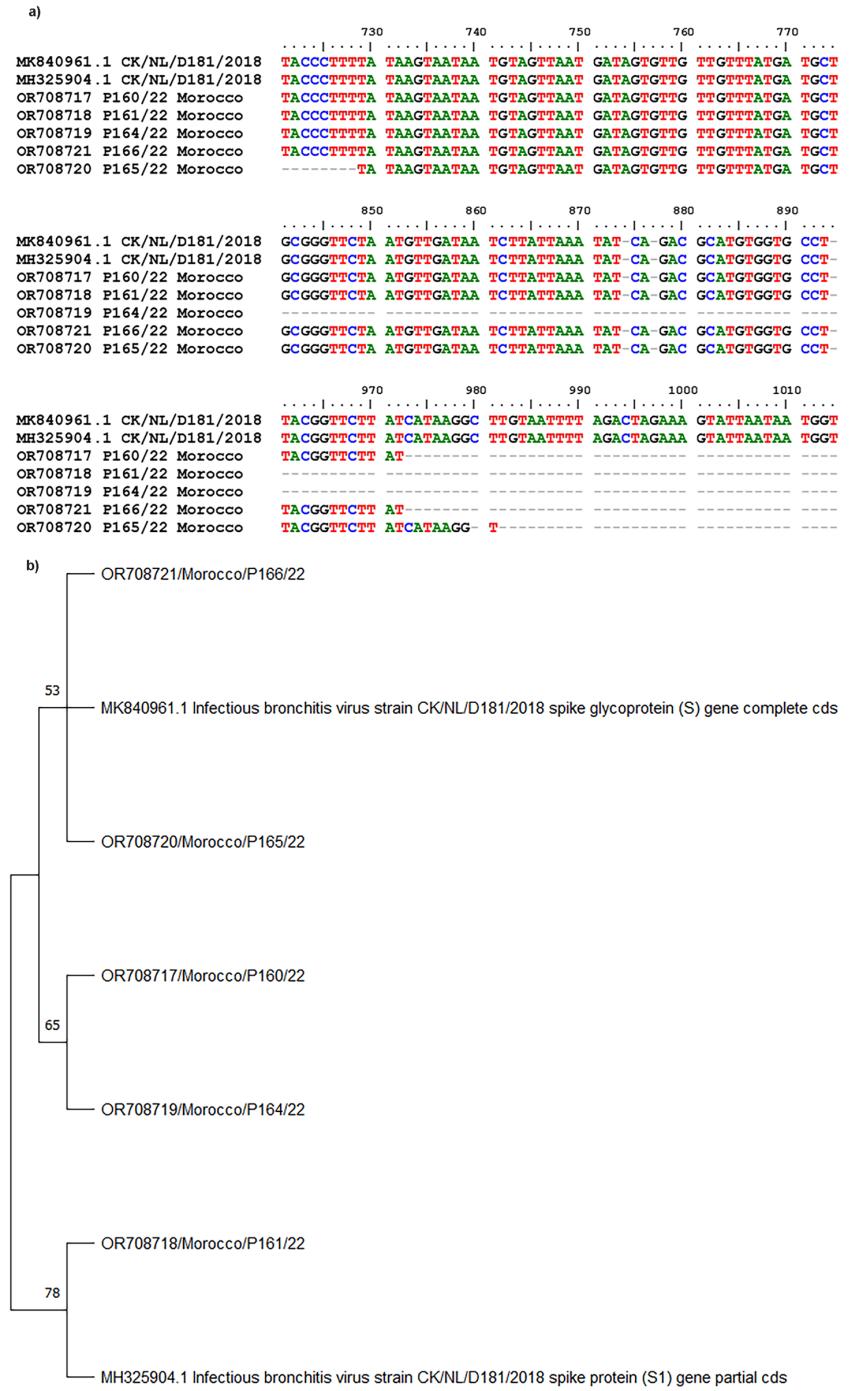
(**a**) Sequence alignment of the 392 bp fragment of the S1 gene of Moroccan IBV D181 strains compared to Dutch strains. (**b**) Phylogenetic tree of the Moroccan strains of IBV genotype D181

Phylogenetic analysis of IBV typically involves comparing the S1 gene nucleotide and protein sequences of different strains [29, 30]. The Moroccan IBV D181 viruses were very closely related to the strain isolated in Dutch layers and breeders in 2018, and closely related to each other (Fig. 5.a). The nucleotide sequence identities among the six Moroccan strains ranged from 98 to 100%. Nucleotide sequence identities reached 90.9–95% with Dutch and Belgian isolates (Fig. 5.b). D181 is considered a new serotype and the second lineage within genotype II (GII) [24, 30].

## Discussion

This study represents the first report of the genotype D181 in poultry production in Morocco. All layer flocks in this study primarily exhibited signs of decreased egg production, accompanied by eggshell abnormalities. The decline in egg production ranged from 6 to 34% and lasted for 2 to 4 weeks. Recovery to normal production was observed in two flocks (F2 and F7), taking 9 to 10 weeks. Respiratory signs were not noted, and mortality rates were unaffected by the infection. Additionally, necropsy results mainly showed an impact on the reproductive tract, specifically lympho-plasmocytic salpingitis, as confirmed by histopathology. Respiratory tract lesions (i.e. fibrinous and caseous sinusitis) were observed in only two flocks (F2 and F3), which tested positive for *Mycoplasma galliseptcium* and *Mycoplasma synoviae* [31, 32]. These findings are in accordance with those of Molenaar et al. [24] who demonstrated, through an experimental challenge, the low pathogenicity of the D181 serotype on the respiratory tract of SPF white layer hens.

However, flock F7 showed mild to moderate lympho-plasmocytic tracheitis with individual cell necrosis, characteristic of classical IBV infection [32], even though respiratory clinical signs were not observed. This may be due to the small sample size of necropsied birds and the inability of workers to detect minor respiratory symptoms on farms. Additionally, unlike other flocks, flock F7 also exhibited abnormal development of the reproductive tract, similar to findings reported by Domanska-Blicharz et al. [33] during IBV episodes caused by the closely related D1466 strain.

On the other hand, broiler flocks displayed elevated mortality rates ranging from 2 to 15%, accompanied by severe respiratory signs. Although molecular screening of other IBV variants, LPAI H9N2, Newcastle disease virus, and AMPV was negative, the possibility of undetected co-infecting pathogens or predisposing environmental factors cannot be ruled out. Molenaar et al. [24] reported similar findings in broiler flocks. Nevertheless, the pathogenicity of genotype D181 on the respiratory tract of broilers cannot be ruled out and requires further investigation.

Molenaar et al. [24] reported a low detection rate of this genotype in broiler farms compared to layers, noting that viruses of the genotype II (D1466, D181) have a tendency for reproductive tract tropism. In contrast, in our study similar numbers of broiler and layer farms were affected with comparable severity of clinical signs. The close proximity of farms in Rabat and Casablanca-Settat, regions with substantial poultry production, could be a contributing factor [34]. These conditions provide an ideal environment for viral and bacterial introduction and spread. The role of broiler farms with loose biosecurity standards in the persistence and spread of avian pathogens in between Moroccan cities cannot be overlooked. The first case in this study involved all flocks of two separate farms located in close proximity, while the second case only affected one out of three flocks on the same farm. This discrepancy may be due to the separation of flocks and more rigorous biosecurity measures applied in the second case compared to the first.

Due to the emergence of its variants, the genome of IBV has been extensively studied in recent years, with a focus on the pathogenic and molecular characterization of this virus due to its genetic diversity [35]. First Identified in the Netherlands, Belgium and Germany, the D181 variant was detected for the first time in Morocco and Africa. This genotype could have been recently introduced to Morocco via international poultry trade with European countries, as this strain is now predominant in layers and breeder flocks in the Netherlands and also has been reported in the United Kingdom and Spain [36]. Another hypothesis is that the virus has been circulating undetected in Moroccan poultry, due to nucleotide mismatches in the primers used [37]. The updated primers used in this study are recommended to enhance the sensitivity of PCR for the detection of this strain [24]. Molecular analysis of IBV D181 has revealed that it is a D1466-like strain, showing 90.9–95% sequence similarity to the D1466 genotype. It is considered as a new serotype and the second lineage within genotype II (GII) [24]. The D181 strain is rapidly spreading in poultry farms worldwide, having evolved from incidental detection in three countries to its isolation in Morocco, where its emergence poses a significant challenge for layer farms. The evolution and spread of this strain are unpredictable, as has been the case with all IBV variants throughout history [38].

In terms of protection, cross-neutralization tests indicate that a single vaccination with a heterologous vaccine of tested IBV serotypes does not provide protection against infection by the D181 strain [24]. Furthermore, the strain D181 showed only 9% cross-neutralization with the closely related D1466 strain (GII), which falls within the same lineage [24, 38].

## Conclusion

We conclude that the IB D181 serotype is circulating in Moroccan poultry and is responsible for significant economic losses. Therefore, it is recommended that this genotype be considered an important differential diagnosis for egg drop in layer flocks and respiratory syndromes with associated mortalities in broilers. Furthermore, ongoing surveillance of IB virus strains in Morocco is essential to monitor its evolution and adjust control measures accordingly.

## Data Availability

No datasets were generated or analysed during the current study.

## Abbreviations

IBV: Infectious bronchitis virus
IB: Infectious bronchitis
RT-PCR: Reverse transcriptase Polymerase chain reaction
SPF: Specific pathogen free chickens
Kb: Kilobases
bp: Base pair
RNA: Ribonucleic acid
DNA: Deoxyribonucleic acid
cDNA: Complementary DNA
Ct: Cycle threshold
